# Effectiveness of Platelet-Rich Plasma Injection for Chronic Achilles Tendinopathy: An Umbrella Systematic Review

**DOI:** 10.7759/cureus.92652

**Published:** 2025-09-18

**Authors:** Nidhin Pallikkara Kuttyadan, Sameer Samad, Momina Shahzad, Kashaf Sanaullah, Syed Hassan M Gillani, Hussnain Mushtaq, Shafeen Bashir Butt, Sardar Khizar Hayat, Muhammad Raif, Syed Momin Ali, Syed Faqeer Hussain Bokhari

**Affiliations:** 1 General Medicine, Kings College Hospital, London, GBR; 2 Biological Sciences, Youngstown State, Youngstown, USA; 3 Medicine, Gujranwala Medical College, Lahore, PAK; 4 Medicine, King Edward Medical University, Lahore, PAK; 5 Internal Medicine, King Edward Medical University, Lahore, PAK; 6 Neurology, Mayo Hospital, Lahore, PAK; 7 Medicine and Surgery, King Edward Medical University, Lahore, PAK; 8 Surgery, King Edward Medical University, Lahore, PAK

**Keywords:** chronic achilles tendinopathy, conservative treatment, meta-analysis, pain management, platelet-rich plasma, sports medicine, systematic review, tendon regeneration, umbrella review, visa-a score

## Abstract

Chronic Achilles tendinopathy (CAT) represents a prevalent overuse injury affecting athletes and active individuals, with conventional treatments showing variable success rates. Platelet-rich plasma (PRP) injection has emerged as a promising biologic therapy, theoretically promoting tendon regeneration through growth factor release. However, clinical evidence regarding PRP effectiveness remains controversial. This umbrella systematic review synthesized findings from systematic reviews and meta-analyses examining PRP injection effectiveness for CAT management. A comprehensive literature search was conducted across five major databases (PubMed, Embase, Scopus, Web of Science, and Cochrane Library) from inception until June 2025. Eight systematic reviews and meta-analyses published between 2018 and 2025 were included, encompassing 170-697 participants across primary studies. Methodological quality was assessed using AMSTAR 2 (A MeaSurement Tool to Assess systematic Reviews) criteria. The predominant finding across reviews was the absence of statistically significant differences between PRP and control interventions for primary outcomes, including pain reduction (visual analog scale) and functional improvement (Victorian Institute of Sport Assessment-Achilles scores). While some reviews reported modest short-term improvements in pain scores at three to 12 weeks, these benefits were neither clinically meaningful nor sustained at longer follow-up periods. Tendon structural outcomes assessed via ultrasonography yielded inconsistent results, with substantial heterogeneity attributed to variability in PRP preparation methods, injection protocols, and outcome measurements. Safety profiles were generally favorable with minimal adverse events. The collective evidence fails to demonstrate PRP superiority over placebo or standard conservative treatments. Current evidence does not support routine PRP use as a first-line treatment for CAT, with established conservative interventions remaining the preferred therapeutic approach.

## Introduction and background

Chronic Achilles tendinopathy (CAT) is a prevalent overuse injury that predominantly affects athletes and physically active individuals. Among runners, between 6% and 10% may be affected by Achilles tendinopathy at any one time, with a lifetime prevalence of up to 52% in middle- and long-distance runners [1]. It primarily involves degeneration of the Achilles tendon due to repetitive mechanical loading, rather than acute inflammation. Patients typically present with posterior heel pain, stiffness during movement, and impaired functional performance. Histopathologically, CAT is characterized by collagen disorganization, increased neovascularization, and tenocyte proliferation, distinguishing it from inflammatory tendinitis and necessitating distinct therapeutic approaches [2]. Despite the significant burden of CAT on quality of life and healthcare systems, its optimal management remains controversial. Conventional conservative treatments such as eccentric loading exercises, orthotics, shockwave therapy, and non-steroidal anti-inflammatory drugs (NSAIDs) have shown variable success [3,4]. While many patients improve with such therapies, a considerable proportion continue to experience persistent pain and functional limitations. This clinical challenge has led to growing interest in biologic therapies that may promote tendon regeneration rather than merely symptom relief.

One such biologic therapy is platelet-rich plasma (PRP) injection, an autologous blood derivative enriched with platelets and associated growth factors [5]. PRP is postulated to enhance tendon healing through the localized release of anabolic cytokines such as platelet-derived growth factor (PDGF), vascular endothelial growth factor (VEGF), and transforming growth factor-beta (TGF-β) [5]. These bioactive molecules are believed to modulate inflammation, stimulate angiogenesis, and promote extracellular matrix remodeling within degenerated tendons. The minimally invasive nature of PRP injection and its potential to address the underlying pathology of CAT have fueled its widespread adoption in musculoskeletal medicine. However, the clinical efficacy of PRP in the treatment of CAT remains contentious. Over the past decade, numerous randomized controlled trials (RCTs), non-randomized studies, and systematic reviews have attempted to evaluate the utility of PRP in CAT with inconsistent findings [6-9]. Some studies suggest significant improvement in pain and function, while others report no benefit over placebo or standard therapy. Variability in PRP preparation methods (e.g., leukocyte-rich vs. leukocyte-poor), dosing regimens, tendon lesion severity, and outcome measurement tools further complicate interpretation. Given these complexities, systematic reviews and meta-analyses have increasingly been conducted to synthesize available evidence and guide clinical decision-making [8,10]. Yet, even among these reviews, conclusions differ, partly due to methodological heterogeneity, small sample sizes, or the inclusion of low-quality primary studies. Some meta-analyses report favorable outcomes with PRP when combined with eccentric training protocols, while others suggest no added benefit over placebo or dry needling [9,11-13]. The conflicting interpretations within these reviews underscore a critical need to evaluate the credibility and consistency of findings across existing systematic reviews.

An umbrella review, also known as a review of systematic reviews, provides a higher level of evidence synthesis by collating, appraising, and integrating findings from multiple systematic reviews on a given topic. This approach is particularly valuable in situations where abundant but inconsistent evidence exists, such as the case of PRP therapy for CAT. Umbrella reviews also enable comparative appraisal of the methodological quality of included reviews, helping to identify areas of consensus, knowledge gaps, and recommendations supported by robust evidence. Accordingly, the objective of this umbrella systematic review is to comprehensively evaluate the effectiveness of PRP injection in the management of CAT by synthesizing the findings from all available systematic reviews and meta-analyses. Specifically, we aim to (1) assess the overall clinical efficacy of PRP in terms of pain reduction and functional improvement, (2) explore the consistency and credibility of findings across reviews, and (3) examine the methodological quality of included systematic reviews using validated appraisal tools. By doing so, we seek to provide clinicians, researchers, and policy makers with a consolidated evidence base to inform future therapeutic decisions, clinical practice guidelines, and research priorities.

In light of the growing demand for biologic therapies in sports and rehabilitation medicine, clarifying the role of PRP in chronic tendon disorders is both timely and necessary. As PRP therapy is not without cost, procedural variation, or potential risks, ensuring its evidence-based use is critical for optimizing patient outcomes and resource allocation. This umbrella review aspires to bridge the evidence gap by offering a bird’s-eye view of the current literature landscape surrounding PRP in CAT.

## Review

Materials and methods

Study Selection

This umbrella systematic review was conducted in accordance with the Preferred Reporting Items for Systematic Reviews and Meta-Analyses (PRISMA) 2020 guidelines and the PRIOR (Preferred Reporting Items for Overviews of Reviews) statement [14,15]. A comprehensive literature search was performed to identify systematic reviews and meta-analyses evaluating the effectiveness of PRP for CAT. The search was designed to capture both qualitative and quantitative syntheses of RCTs or controlled studies examining PRP as a therapeutic intervention for CAT. Five major databases, PubMed, Embase, Scopus, Web of Science, and the Cochrane Library, were systematically searched from inception until June 25, 2025. The search strategy included a combination of Medical Subject Headings (MeSH) and relevant keywords such as “platelet-rich plasma,” “PRP,” “Achilles tendinopathy,” “Achilles tendonitis,” “systematic review,” and “meta-analysis.” No language restrictions were applied. Reference lists of all included articles were also manually screened to identify additional eligible reviews. All search results were imported into Zotero for deduplication, and the deduplicated records were uploaded to the Rayyan platform for screening. Two reviewers independently assessed the titles and abstracts to determine potential eligibility. Full-text versions of potentially relevant articles were retrieved for detailed evaluation. Disagreements between reviewers regarding inclusion were resolved through discussion and, if necessary, arbitration by a third reviewer. The selection process was documented using a PRISMA flow diagram, which outlined the number of records identified, screened, excluded, and ultimately included in the final analysis.

Eligibility Criteria

Eligible studies included systematic reviews or meta-analyses that examined the efficacy of PRP injections for the management of CAT in adults. To be considered, reviews must have included at least one RCT or comparative study involving human participants aged 18 years or older with a clinical or imaging-confirmed diagnosis of CAT. PRP intervention was defined broadly, including single or multiple injections, leukocyte-rich or leukocyte-poor formulations, and variable dosing or administration techniques. Reviews that compared PRP to placebo, no treatment, eccentric loading protocols, dry needling, corticosteroid injection, or other conservative interventions were considered eligible. Only reviews that explicitly evaluated clinical outcomes such as pain reduction, functional improvement, imaging changes, return to sport, or adverse events were included. Reviews that combined Achilles tendinopathy with other tendinopathies without presenting disaggregated data were excluded. Narrative reviews, scoping reviews, conference abstracts, and reviews without a systematic methodology or without a clearly defined literature search were excluded. Articles that focused solely on preclinical, animal, or in vitro studies were also excluded. Gray literature was also excluded.

Data Extraction

Data were independently extracted from each included review by two reviewers using a pre-designed and standardized data extraction form. The form included variables such as first author, year of publication, journal, number and type of included primary studies, and total sample size. Additional data included comparator interventions, follow-up durations, and reported outcomes. For reviews with meta-analyses, extracted data also included effect sizes (e.g., mean difference and standardized mean difference), confidence intervals, p-values, I² values for heterogeneity, and details of subgroup or sensitivity analyses if performed. Any discrepancies in data extraction were resolved through discussion or with input from a third reviewer.

Quality Assessment

The methodological quality of all included systematic reviews and meta-analyses was assessed using the AMSTAR 2 (A Measurement Tool to Assess Systematic Reviews) checklist. This tool evaluates the quality of systematic reviews across 16 key domains, including protocol registration, adequacy of the literature search, risk of bias assessment of included studies, meta-analytic methods, and assessment of publication bias. Each review was independently rated by two reviewers as high, moderate, low, or critically low quality based on the presence or absence of critical and non-critical flaws as outlined in the AMSTAR 2 guidance. Any disagreements in scoring were resolved through discussion and consensus.

Data Analysis

Given that this umbrella review included previously published meta-analyses and systematic reviews, a meta-analysis was not conducted. Instead, a narrative synthesis of the findings across reviews was performed. The synthesis focused on identifying areas of consensus and divergence in the reported effectiveness of PRP injections for CAT. The direction, strength, and consistency of treatment effects across reviews were assessed and contextualized alongside heterogeneity estimates and methodological quality.

Results

Study Selection Process

**Figure 1:**
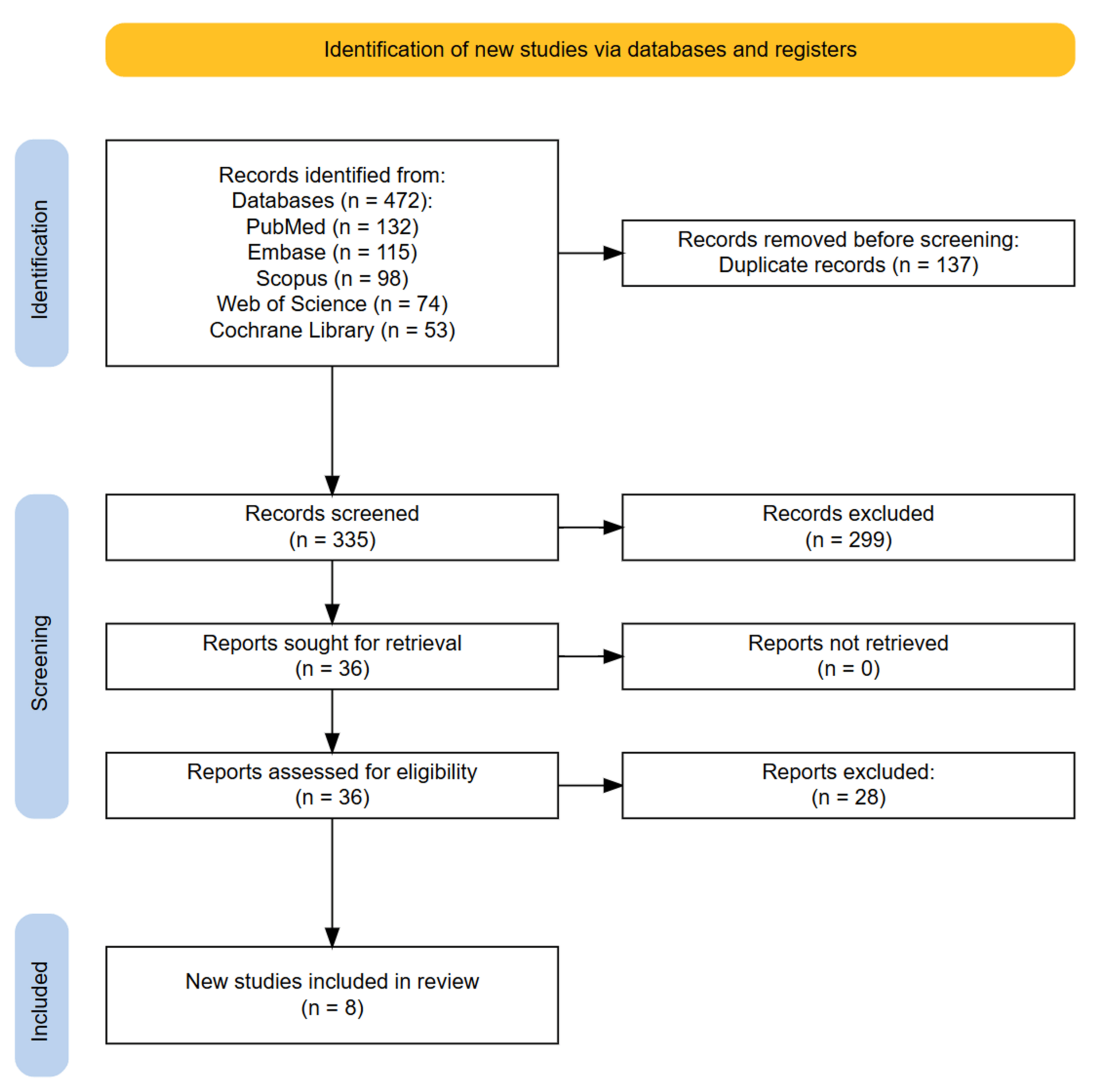
PRISMA diagram illustrating the study selection process. PRISMA, Preferred Reporting Items for Systematic Reviews and Meta-Analyses

A total of 472 records were initially retrieved through systematic searches across PubMed, Embase, Scopus, Web of Science, and the Cochrane Library. After the removal of 137 duplicate entries using Zotero, 335 unique records remained for screening. These records were independently reviewed by two researchers based on titles and abstracts using the Rayyan web application. Following the initial screening phase, 36 articles were identified as potentially eligible and were retrieved for full-text evaluation. Upon thorough assessment against the predefined inclusion and exclusion criteria, eight systematic reviews and meta-analyses met all eligibility requirements and were included in the final umbrella review. The remaining 28 full-text articles were excluded for various reasons, including ineligible population (n=10), absence of PRP-specific subgroup analysis for Achilles tendinopathy (n=10), lack of quantitative synthesis or systematic methodology (n=7), and insufficient outcome data related to pain or function (n=1). No additional studies were identified through manual screening of the reference lists of the included reviews or related publications. Any disagreements between reviewers during the screening and selection process were resolved through consensus, and no arbitration by a third reviewer was required. The entire selection process is summarized in the PRISMA flow diagram (Figure 1).

Study Characteristics

Based on the included systematic reviews and meta-analyses, several key study characteristics emerge regarding PRP effectiveness for CAT. Eight reviews published between 2018 and 2025 were included, with sample sizes ranging from 170 to 697 participants across primary studies [8-13,16,17]. The reviews predominantly analyzed RCTs, with some including prospective cohorts and retrospective studies. All reviews focused on adult populations (≥18 years) with clinically or imaging-confirmed CAT, including both mid-portion and insertional variants. PRP interventions varied considerably across studies in terms of preparation methods (leukocyte-rich vs. leukocyte-poor), injection techniques, and dosing regimens. Control groups typically involved placebo injections, though some studies compared PRP to eccentric training protocols or other conservative treatments. Primary outcomes consistently included pain reduction measured by the visual analog scale (VAS) and functional improvement assessed using the Victorian Institute of Sport Assessment-Achilles (VISA-A) questionnaire. Secondary outcomes encompassed tendon structural changes via ultrasonography, return-to-sport rates, patient satisfaction, and adverse events. Follow-up periods ranged from six weeks to 54 months, with most studies reporting short-term (three months), intermediate-term (six months), and long-term (12+ months) outcomes (Table 1).

**Table 1:** A summary of the study characteristics of the included studies. RCTs, randomized controlled trials; CAT, chronic Achilles tendinopathy; ATR, Achilles tendon rupture; PRP, platelet-rich plasma; VISA-A, Victorian Institute of Sport Assessment-Achilles; VAS, visual analog scale; NPRS, numeric pain rating scale; ARTS, Achilles tendon rupture score; RoB 2.0, Risk of Bias tool version 2.0

Author	Year	Journal	Included Studies	Sample Size	Type of Studies Included	Aim	Population	Intervention	Control	Outcomes	Follow-Up	Methodological Quality & Tools
Ali Elsiddig Ahmed et al. [9]	2025	Cureus	13	697	5 RCTs, 5 prospective cohorts, 2 retrospective studies, 1 cross-sectional study	To evaluate the effectiveness of PRP in reducing pain, improving function, and facilitating recovery in CAT	Adults ≥18 years with clinically and/or imaging-confirmed CAT (symptoms >6 weeks), including mid-portion and insertional tendinopathy	PRP injections (any preparation method, injection technique, number of injections)	Placebo	Pain reduction (VAS/NPRS), functional improvement (VISA-A), return to activity, patient satisfaction, safety	4 weeks to 54 mo	Newcastle-Ottawa scale
Ling et al. [12]	2024	The Orthopaedic Journal of Sports Medicine	6	422	RCTs	To pool the available data and evaluate the evidence of the effect of PRP injections on CAT	Clinical or ultrasonographic diagnosis of CAT	PRP injection	Placebo	Primary: VISA-A scores and maximal Achilles tendinopathy thickness on ultrasound; secondary: patient satisfaction and adverse events	Short term: 3 mo; intermediate term: 6 mo; long term: 12 mo	RoB 2.0 tool
Arthur Vithran et al. [8]	2023	Journal of Clinical Medicine	8	526	RCTs	To investigate whether PRP injections can improve outcomes in patients with CAT	Patients diagnosed with CAT	Non-surgical treatment with local PRP injection	Placebo	VISA-A score, Achilles tendon thickness, VAS pain score, patient satisfaction, return to exercise/sport	6 weeks to 48 weeks	Modified Jadad scale
Huang et al. [11]	2023	World Journal of Orthopedics	8 CAT studies, 5 ATR studies	8 RCTs for CAT, 5 RCTs for ATR	RCTs	To assess PRP's effectiveness in treating ATR and CAT	Patients with CAT or ATR	Local PRP injection	Placebo	VISA-A score, VAS for pain, Achilles tendon thickness changes, return to sport rates, patient satisfaction, ankle flexion, heel-rise height, adverse events	6 weeks to 24 mo	Modified Jadad scale
Nauwelaers et al. [17]	2020	Foot and Ankle Surgery	4	170	RCTs	To establish the existing evidence of PRP injections for CAT on the functional outcome	Diagnosis of mid-portion CAT	PRP injection	Placebo	Primary: VISA-A score at 3, 6, and 12 mo; secondary: tendon structure changes via ultrasonography	3 mo, 6 mo, and 12 mo post-injection	RoB 2.0 tool
Chen et al. [16]	2021	Platelets	11	574 (225 AT, 349 ATR)	RCTs	To evaluate whether PRP improves the outcomes of ATR or CAT	Patients with CAT or ATR	PRP injection	Placebo	Primary: VISA-A, ARTS score improvement; secondary: Tendon thickness, VAS pain, heel-rise work, maximum heel-rise height, dorsal/plantar flexion, return to sports, complications	6 weeks to 24 mo	RoB 2.0 tool
Liu et al. [13]	2018	Medicine	5	189	RCTs	To evaluate the current evidence for the efficacy of PRP as a treatment for CAT	Diagnosis of CAT	Injection of PRP around the tendon	Placebo	Primary: VISA-A; secondary: VAS, Achilles tendon thickness (ultrasonography)	6 weeks to 1 year (6, 12, 24 weeks, 1 year)	RoB 2.0 tool
Zhang et al. [10]	2018	Clinical Orthopaedics and Related Research	4	170	RCTs	To determine PRP plus eccentric strength training effectiveness in CAT	Adults with CAT	PRP injection with eccentric training	Placebo	Primary: VISA-A score; secondary: tendon thickness, color Doppler activity, pain, return to sports activity	3 to 12 mo	RoB 2.0 tool, Modified Coleman methodology score

The main findings across the eight systematic reviews revealed inconsistent evidence for PRP effectiveness in CAT. Most reviews found no significant differences between PRP and placebo for pain reduction (VAS scores) or functional improvement (VISA-A scores) at multiple time points. While some studies reported short-term benefits at three to 12 weeks, these effects were not sustained long-term [11,13,16]. Tendon structural changes showed inconsistent results, with no clear pattern of improvement. Return-to-sport rates and patient satisfaction showed minimal differences between PRP and control groups. High heterogeneity was noted across studies, attributed to variability in PRP preparation methods, injection protocols, and outcome measurements. Safety profiles were generally favorable, with minimal adverse events reported, primarily consisting of mild injection-site reactions. Overall, the reviews concluded there is insufficient evidence to support PRP as superior to placebo or standard conservative treatments for CAT (Table 2).

**Table 2:** A summary of the main findings of the included studies. MD, mean difference; WMD, weighted mean difference; CI, confidence interval; SMD, standardized mean difference; RR, relative risk; VISA-A, Victorian Institute of Sport Assessment-Achilles; VAS, visual analog scale; I², heterogeneity statistic; P, P-value

Author	Pain Outcome	Functional Outcome	Tendon Structure Outcomes	Return to Sport	Adverse Events	Summary	Conclusions
Ali Elsiddig Ahmed et al., 2025 [9]	Pooled mean VAS: 71.24 (95% CI: 53.06-89.42)	Pooled mean VISA-A: 71.24 (95% CI: 53.06-89.42)	Not reported	85% of patients returned to activity (95% CI: 65-98%)	Not reported	PRP demonstrated significant pain reduction and functional improvement in CAT, high heterogeneity (I²=97%) was noted across studies, likely due to variability in PRP preparation and treatment protocols, 85% return to activity rate and 72% patient satisfaction	PRP offers promise for CAT with evidence of pain relief and functional improvement; however, variability in outcomes emphasizes the need for standardized approaches and further research to define its clinical role, leukocyte-poor PRP may be preferable to leukocyte-rich formulations
Ling et al., 2024 [12]	VISA-A scores, short term: MD 2.28 (95% CI: -1.95 to 6.51), P=0.29, intermediate term: MD 1.83 (95% CI: -2.66 to 6.32), P=0.42, Long term: MD 3.46 (95% CI: -8.62 to 15.55), P=0.57	Same as pain outcome, VISA-A covers pain, function, and participation domains in 8 questions (0=highest severity, 100=lowest severity)	Maximal Achilles tendinopathy thickness, short term: MD 0.26 mm (95% CI: -0.71 to 1.24), P=0.60, intermediate term: MD -0.84 mm (95% CI: -2.12 to 0.43), P=0.20, long term: MD -0.28 mm (95% CI: -1.19 to 0.64), P=0.55	Not reported	No infections, hematomas, or ruptures reported except 2 participants required surgical intervention due to severe pain, common mild side effects included bleeding, bruising, swelling, mild discomfort at injection site, few side effects lasted 6 mo	No significant differences between PRP injection and control groups for both VISA-A scores and maximal Achilles tendinopathy thickness at any time point	No solid evidence has been established for PRP effectiveness in CAT despite increasing popularity, heterogeneity of tendinopathy pathology and PRP methodology should be controlled by better-designed trials, further research needed before recommending as standard treatment
Arthur Vithran et al., 2023 [8]	VAS (0-100 scale), no significant difference at 6 weeks (MD=6.75, P=0.30) and 24 weeks (MD=10.46, P=0.11), significant improvement at 12 weeks (MD=11.30, P<0.00001)	VISA-A score, PRP group slightly higher than placebo at 6 weeks (MD=1.92), 12 weeks (MD=0.20), and 24 weeks (MD=2.75), differences not statistically significant (P=0.27)	Achilles tendon thickness, higher in PRP group at 12 weeks (MD=0.34, 95% CI -0.04 to 0.71, P=0.08), not statistically significant	Return to exercise rate higher in PRP group (RR=1.11, 95% CI 0.87-1.42, P=0.40), not statistically significant	No adverse reactions reported after tendon PRP injection	Moderate evidence that PRP injection did not significantly improve clinical outcomes compared to placebo for most measures, except VAS improvement at 12 weeks	There is no proof that PRP injections can enhance patient functional and clinical outcomes for CAT, more rigorous designs and standardized clinical RCTs are needed, augmenting frequency of PRP injections may boost outcomes
Huang et al., 2023 [11]	CAT: VAS improved at 3 mo (WMD=11.30, 95% CI: 7.33-15.27, significant), no difference at 6 weeks or 6 mo, ATR: no significant differences at any time point	CAT: VISA-A no significant differences at 6 weeks, 3 mo, 6 mo, ATR: VISA-A no significant differences at 3, 6, 12 mo	CAT: tendon thickness no difference (WMD=0.34, 95% CI: -0.04-0.71), ATR: ankle mobility improved at 12 mo only (WMD=-0.98, 95% CI: -1.41 to -0.56, significant)	CAT: No difference in return to sport (WMD=1.11, 95% CI: 0.87-1.42), ATR: No difference (WMD=1.20, 95% CI: 0.77-1.87)	No significant differences in adverse events between groups (WMD=0.85, 95% CI: 0.50-1.45), events included re-rupture, infection, pain	CAT: PRP improved immediate VAS scores at 3 mo but not VISA-A, tendon thickness, satisfaction, or return to sport, ATR: PRP improved only long-term ankle mobility at 12 mo, no other benefits	This study showed no significant efficacy of PRP injection alone in patients with ATR and CAT, authors recommend larger studies with standardized PRP preparation and exploring combination therapies
Nauwelaers et al., 2020 [17]	No significant differences at any time point	No significant differences at any time point	Inconsistent findings: significant reduction in tendon thickness and vascularity in one study, increased tendon thickness in others, no difference between groups in third study	Not reported	Safety profile: no complications or serious side effects were reported, minor effects included injection-related pain mentioned in some studies	No significant difference in VISA-A score between PRP and placebo groups at 3, 6, or 12 mo, results were heterogeneous (I² >50%), tendon structure changes were inconsistent across studies	PRP has no clear additional value in the management of midsubstance CAT and therefore should not be used as a first-line treatment option, treatment should primarily be based on self-management advice, education, and exercise therapy, need for standardized PRP preparation and administration protocols
Chen et al., 2021 [16]	VAS (0-100 scale) at 6 weeks, 3 mo, and 6 mo, CAT patients: significant improvement at 3 mo only (MD=11.30, 95% CI=7.33-15.27, P<0.00001)	VISA-A score (CAT patients): 6 weeks: significant improvement in the PRP group (MD=2.64, 95% CI=1.12-4.15, P=0.0006), 3 and 6 mo: no significant difference, ATRS score (ATR patients): no significant differences at 3, 6, or 12 mo	Tendon thickness (CAT patients): No significant difference at 3 mo (MD=0.34, 95% CI=-0.04 to 0.71, P=0.08)	Return to sports at 6 weeks: no significant difference (RR=1.20, 95% CI=0.77-1.87, P=0.42)	Re-rupture rate: no significant difference (RR=1.22), infection rate: no significant difference (RR=0.15), pain/discomfort: no significant difference (RR=1.02)	Minimal evidence supporting PRP injection for CAT or ATR, only short-term benefits were observed for CAT patients at 6 weeks for VISA-A scores and 3 mo for pain, no functional or structural improvements demonstrated for ATR patients	No evidence indicates that PRP injection can improve patient-reported, clinical, or functional outcomes of CAT or ATR, PRP injection offers no patient benefit and is not superior to control treatments, authors hypothesized that increasing injection frequency might improve outcomes, requiring further RCTs to verify
Liu et al., 2018 [13]	VAS (0-100 scale) 6 weeks: SMD=1.35, not significant, 12 weeks: SMD=1.10, significant improvement, 24 weeks: SMD=1.48, not significant	VISA-A (0-100 scale) 6 weeks: SMD=0.46, significant improvement, 12 weeks: SMD=0.20, not significant, 24 weeks: SMD=0.77, not significant, 1 year: SMD=0.83, not significant	Achilles tendon thickness (ultrasonography) 12 weeks: SMD=1.51, significantly thinner in PRP group	Not reported	No adverse reactions reported after PRP injection	PRP showed limited superior effects compared to placebo, only significant improvements at 6 weeks for VISA-A, 12 weeks for VAS, and tendon thickness reduction, high heterogeneity between studies was noted, particularly due to multiple injections vs a single injection	PRP injection around the Achilles tendon is an option for treatment of CAT, limited evidence supports that PRP is not superior to placebo treatment, results require verification by large, well-designed, homogeneous RCTs
Zhang et al., 2018 [10]	Pain at rest and pain while walking were assessed on a 0-10 numeric rating scale, mixed results	VISA-A score (0-100 points, higher scores = better function), mean difference: 5.3 (95% CI: -0.7 to 11.3, P=0.085), no significant difference between groups	Tendon thickness change measured by ultrasound, MD: 0.2 mm (95% CI: -0.6 to 1.0 mm, P=0.663), color Doppler activity (Grade 0-4 scale), MD: 0.1 (95% CI: -0.7 to 0.4, P=0.695), no significant differences	Two studies evaluated return to sport, no significant differences	Not reported	No significant differences between PRP and saline injection for any primary or secondary outcomes in CAT when combined with eccentric training	PRP injection with eccentric training did not improve VISA-A scores, reduce tendon thickness, or reduce color Doppler activity compared with saline injection with eccentric training, larger RCTs are needed to confirm results, PRP cannot be recommended for general use until a clear benefit is demonstrated

Quality Assessment

The methodological quality assessment using AMSTAR 2 revealed high quality across the eight included systematic reviews. All reviews demonstrated protocol registration, adequate risk of bias assessment of primary studies, and appropriate evaluation of publication bias. Conflicts of interest were declared in all included studies. All of the included reviews achieved high-quality ratings, demonstrating comprehensive literature search strategies and appropriate statistical methods. Overall, the consistent quality across reviews strengthens confidence in the synthesized findings regarding PRP effectiveness for CAT.

Discussion

This umbrella systematic review synthesized findings from eight systematic reviews and meta-analyses examining the effectiveness of PRP injection for CAT, revealing a complex and largely inconclusive evidence landscape. Despite the theoretical biological rationale and widespread clinical adoption of PRP therapy, the collective evidence fails to demonstrate consistent superiority over placebo or standard conservative treatments across multiple clinical domains. The predominant finding across reviews was the absence of statistically significant differences between PRP and control interventions for primary outcomes, including pain reduction and functional improvement. This lack of efficacy was particularly evident in the VISA-A scores, which serve as the gold standard for functional assessment in Achilles tendinopathy [18]. While some reviews reported modest short-term improvements in pain scores at three to 12 weeks, these benefits were neither clinically meaningful nor sustained at longer follow-up periods [11,13,16]. The transient nature of any observed benefits raises questions about the durability of PRP regenerative effects and suggests that any improvements may be attributable to the injection procedure itself rather than the biological activity of PDGFs.

The inconsistency in tendon structural outcomes further undermines the case for PRP efficacy. Ultrasonographic assessments of tendon thickness and vascularity yielded conflicting results across studies, with some showing marginal improvements while others demonstrated no changes or even worsening of structural parameters [10,13,17]. This variability is particularly concerning given that tendon regeneration and remodeling represent the primary theoretical mechanism through which PRP is hypothesized to exert its therapeutic effects. The absence of consistent structural improvements suggests that PRP may not be effectively modulating the underlying pathophysiology of chronic tendinopathy, which is characterized by collagen disorganization, neovascularization, and tenocyte proliferation.

Several factors may contribute to the disappointing clinical results observed across reviews. First, the substantial heterogeneity in PRP preparation methods represents a critical confounding variable. The included studies employed diverse protocols regarding platelet concentration, leukocyte content, activation methods, and storage conditions, all of which can significantly impact the bioavailability and activity of growth factors [19]. The debate between leukocyte-rich and leukocyte-poor formulations remains unresolved, with some evidence suggesting that excessive leukocyte content may promote inflammatory responses that counteract the intended regenerative effects [20]. Additionally, the lack of standardized injection techniques, including needle gauge, injection volume, and anatomical targeting, may have influenced treatment outcomes across studies.

The chronic nature of Achilles tendinopathy itself may also limit the therapeutic potential of PRP intervention. Unlike acute injuries where PDGFs can facilitate normal healing cascades, chronic tendinopathy involves established degenerative changes with altered cellular metabolism and compromised vascular supply [2]. The microenvironment within chronically degenerated tendons may be inherently resistant to growth factor stimulation, potentially explaining why PRP shows promise in acute soft tissue injuries but fails to demonstrate consistent benefits in chronic tendinopathies. Furthermore, the placebo effect associated with injection procedures cannot be overlooked. Several reviews noted that both PRP and placebo groups showed improvements from baseline, suggesting that the act of injection itself, combined with associated patient expectations and concurrent rehabilitation protocols, may contribute to symptom improvement independent of the injected substance [9,13]. This phenomenon is particularly relevant in chronic pain conditions where psychological factors play a significant role in symptom perception and functional capacity.

The economic implications of these findings are substantial. PRP therapy involves considerable costs related to blood collection, processing equipment, and specialized personnel, while the procedure carries inherent risks including infection, nerve injury, and temporary pain exacerbation [21]. Given the lack of demonstrated superiority over conservative treatments such as eccentric loading exercises, the routine use of PRP for CAT cannot be justified from a cost-effectiveness perspective.

The findings of this umbrella review align with broader trends in regenerative medicine, where promising preclinical results often fail to translate into clinically meaningful outcomes. The complexity of human tendon pathophysiology, combined with the challenges of standardizing biological therapies, highlights the need for more rigorous research methodologies and realistic expectations regarding the therapeutic potential of autologous blood-derived treatments.

Limitations and future directions

This umbrella review has several limitations that warrant acknowledgment. The heterogeneity in primary study methodologies, PRP preparation protocols, and outcome measurement tools limited the ability to draw definitive conclusions about specific PRP formulations or injection techniques. The relatively small sample sizes in many primary studies may have been insufficient to detect clinically meaningful differences, particularly given the variable natural history of CAT. Additionally, the review was limited to published systematic reviews and meta-analyses, potentially excluding more recent primary studies that could provide updated evidence.

Future research should prioritize the development of standardized PRP preparation and administration protocols to enable meaningful comparison across studies. Large-scale, multicenter RCTs with adequate statistical power are needed to definitively establish the role of PRP in chronic tendinopathy management. Studies should incorporate advanced imaging techniques, biomarker analysis, and patient-specific factors to identify potential responders and optimize treatment protocols. Additionally, cost-effectiveness analyses comparing PRP to established conservative treatments would provide valuable guidance for clinical decision-making and healthcare resource allocation. Until such evidence emerges, clinicians should prioritize proven conservative interventions, including eccentric loading exercises, patient education, and load management strategies, as first-line treatments for CAT.

## Conclusions

This umbrella systematic review demonstrates that current evidence does not support the routine use of PRP injection as a superior treatment for CAT compared to placebo or standard conservative therapies. Despite theoretical biological rationale and widespread clinical adoption, eight systematic reviews consistently failed to show sustained clinical benefits in pain reduction, functional improvement, or tendon structural outcomes. The substantial heterogeneity in PRP preparation methods, injection protocols, and study methodologies limits definitive conclusions about specific formulations. Given the considerable costs and procedural risks associated with PRP therapy, clinicians should prioritize proven conservative interventions, including eccentric loading exercises, patient education, and load management strategies, as first-line treatments. Future research should focus on standardized PRP protocols, larger multicenter trials, and cost-effectiveness analyses to definitively establish the role of PRP in chronic tendinopathy management before recommending its widespread clinical implementation.
